# Machine and Deep Learning Based Radiomics Models for Preoperative Prediction of Benign and Malignant Sacral Tumors

**DOI:** 10.3389/fonc.2020.564725

**Published:** 2020-10-16

**Authors:** Ping Yin, Ning Mao, Hao Chen, Chao Sun, Sicong Wang, Xia Liu, Nan Hong

**Affiliations:** ^1^ Department of Radiology, Peking University People’s Hospital, Beijing, Beijing Municipality, China; ^2^ Department of Radiology, Yantai Yuhuangding Hospital, Qingdao University, Yantai, China; ^3^ Pharmaceutical Diagnostics, GE Healthcare, Shanghai, China

**Keywords:** deep learning, radiomics, sacral tumors, machine learning, computed tomography

## Abstract

**Purpose:**

To assess the performance of deep neural network (DNN) and machine learning based radiomics on 3D computed tomography (CT) and clinical characteristics to predict benign or malignant sacral tumors.

**Materials and methods:**

This single-center retrospective analysis included 459 patients with pathologically proven sacral tumors. After semi-automatic segmentation, 1,316 hand-crafted radiomics features of each patient were extracted. All models were built on training set (321 patients) and tested on validation set (138 patients). A DNN model and four machine learning classifiers (logistic regression [LR], random forest [RF], support vector machine [SVM] and k-nearest neighbor [KNN]) based on CT features and clinical characteristics were built, respectively. The area under the receiver operating characteristic curve (AUC) and accuracy (ACC) were used to evaluate different models.

**Results:**

In total, 459 patients (255 males, 204 females; mean age of 42.1 ± 17.8 years, range 4–82 years) were enrolled in this study, including 206 cases of benign tumor and 253 cases of malignant tumor. The sex, age and tumor size had significant differences between the benign tumors and malignant tumors (*χ^2^_sex_* = 10.854, *Z_age_* = −6.616, *Z_size_* = 2.843, *P* < 0.05). The radscore, sex, and age were important indicators for differentiating benign and malignant sacral tumors (odds ratio [OR]1 = 2.492, OR2 = 2.236, OR3 = 1.037, *P* < 0.01). Among the four clinical-radiomics models (RMs), clinical-LR had the best performance in the validation set (AUC = 0.84, ACC = 0.81). The clinical-DNN model also achieved a high performance (an AUC of 0.83 and an ACC of 0.76 in the validation set) in identifying benign and malignant sacral tumors.

**Conclusions:**

Both the clinical-LR and clinical-DNN models would have a high impact on assisting radiologists in their clinical diagnosis of sacral tumors.

## Introduction

Although sacral tumors are rare, all components of sacrum can give rise to benign or malignant tumors (1, 2). Given the prominent hematopoietic function of the sacrum, it is one of the most common sites for bone metastatic tumors (3). Primary malignant bone tumors of the sacrum include chordoma, myeloma, lymphoma, chondrosarcoma, osteosarcoma, and Ewing’s sarcoma, teratoma, etc. Chordoma is the most common primary malignant tumors of the sacrum, accounting for about 40% of all primary tumors (4, 5). Benign tumors mainly include giant cell tumors (GCTs), schwannoma, neurofibroma, aneurysmal bone cysts, bone cyst, cavernous hemangioma, solitary fibroma, osteoid osteoma, and osteoblastoma, etc. Among them, GCTs are the most common, accounting for about 13% (4).

Sacral tumors are often difficult to diagnose due to overlapping clinical symptoms, diverse pathologic findings, and complex imaging features (6). Besides, the treatment of sacral tumors is often a challenging process and varies in approach. For all primary malignant sacral tumors and benign lesions involving lower segments when preservation of both S3 roots is possible, wide resection should be selected. Serial embolization may be worthwhile for benign sacral tumors that extend above S3 (7). Accurate preoperative identification of benign or malignant sacral tumors is essential for individualized treatment. Since sacral tumors are rare and similar on conventional imaging, a noninvasive and highly accurate preoperative diagnostic tool is needed for radiologists.

Machine learning-based tools have developed rapidly in medical imaging in recent years, especially in oncology. Various machine learning algorithms have been applied to create decision models that aid in clinical diagnosis and treatment (8, 9). Few recent studies have used radiomics analysis to identify sacral tumors with a relatively small sample size (1, 5, 10). Yin et al. (1) compared three different feature selection methods and three machine learning classifiers to identify primary sacral chordoma and GCT based on computed tomography (CT) features. Their study demonstrated that the least absolute shrinkage and selection operator (LASSO) + generalized linear models perform best. Deep neural network (DNN), as a deep architecture, has shown excellent performance in classification tasks and is increasingly being used in various areas of cancer research (11, 12). Early studies on the application of deep learning to the detection or classification of lesions have shown that it performs better than traditional techniques and even better than radiologists on some tasks (13–18). Ren et al. (19) proposed a novel manifold regularized classification DNN to enhance CT image-based lung nodule classification. Feng et al. (20) developed an end-to-end DNN model that can achieve promising performance in breast cancer cell nuclei classification. Considering the fact that deep learning requires a larger sample size than radiomics, we were interested to find out how these machine and deep learning algorithms performed to identify benign and malignant sacral tumors based on our relatively large sample size.

Therefore, the aim of our study was to determine the performance of DNN and four machine learning classifiers (logistic regression [LR], random forest [RF], support vector machine [SVM] and k-nearest neighbor [KNN]) based on CT features and clinical characteristics to predict benign or malignant sacral tumors.

## Materials and Methods

### Patients and Data Acquisition

This single center retrospective study was approved by our local ethics committee and waived written informed consent. A total of 505 patients with pathologically confirmed sacral tumors in our institution from January 2007 to December 2019 were retrospectively analyzed. All patients had a single sacral tumor that was detected on CT within 1 month before the initial surgery. Patients had sacral tumors without preoperative CT images (n = 41), or with obvious artifacts (n = 5) were excluded. Finally, a total of 459 patients with sacral tumor were included in the study. Sex, age and maximal tumor size of patients were also analyzed.

All CT images were acquired on each patient using multi-detector row CT systems (Philips iCT 256, Philips Medical System; GE Lightspeed VCT 64, GE Medical System). The acquisition parameters were as follows: 120 kV, 685 mAs, slice thickness = 5 mm, matrix = 512 × 512 mm, field of view = 350 × 350 mm. The CT images were reconstructed with a standard kernel.

### Tumor Segmentation

MITK software version 2018.04.2 (www.mitk.org) was used for the semi-automatic segmentation of all tumors (21). First, we manually delineated the edge of the lesion at the axial, sagittal, and coronal sites, respectively. Then, a three-dimensional lesion was automatically formed and manually corrected by a musculoskeletal radiologist with 5 years of experience and a senior musculoskeletal radiologist with 20 years of experience.

### Feature Extraction and Selection

In total, 1,316 radiomics features of each patient were extracted from the CT images using the Artificial Intelligence Kit software version 3.3.0 (GE Healthcare, China) based on the open-source Pyradiomics python package, which including 18 first-order histogram features, 24 gray-level co-occurrence matrix features, 14 shape features, 14 gray-level dependence matrix features, 16 gray-level size-zone matrix features, 16 gray-level run-length matrix features, 744 wavelet features, 5 neighboring gray-tone difference matrix features, 186 Laplacian of Gaussian (LoG_sigma=2.0/3.0_) features, and 279 local binary pattern features.

We preprocessed the data and normalized the extracted features. When the data value exceeded the range of mean value and standard deviation, the median of specific variance vector was used to replace the outliers. In addition, we standardized the data in a specific interval. The consistency of features from different machines was evaluated by using intra- and interclass correlation coefficients (ICC). An ICC greater than 0.75 was considered as good agreement.

To reduce overfitting or selection bias in our radiomics model, we used minimum redundancy maximum relevance (mRMR) and LASSO to select the features. At first, mRMR was performed to eliminate redundant and irrelevant features, and 20 features were retained. Then, LASSO was conducted to choose the optimized subset of features. After the number of features was determined, the most predictive radiomics features were chosen to construct the final model.

### Model Building and Validation

First, we randomly divided the patients into the training (n = 321) and validation (n = 138) sets by a ratio of 7:3. Then, we built four different radiomics models (RMs) by using LR, RF, SVM, and KNN. Finally, we also built a DNN model based on selected features with a hidden layer number of 3. The number of hidden layer nodes in each layer is 4, 3, and 2, respectively.

Clinical features were compared *via* univariate analysis, and variables with *P* value < 0.05 were included in the clinical model. When combined RMs and DNN with clinical data, we also constructed the clinical-RMs and clinical-DNN model. Models were trained with the training set by using the repeated 10-fold cross-validation method, and estimation performance was evaluated with the validation set.

**Figure 1:**
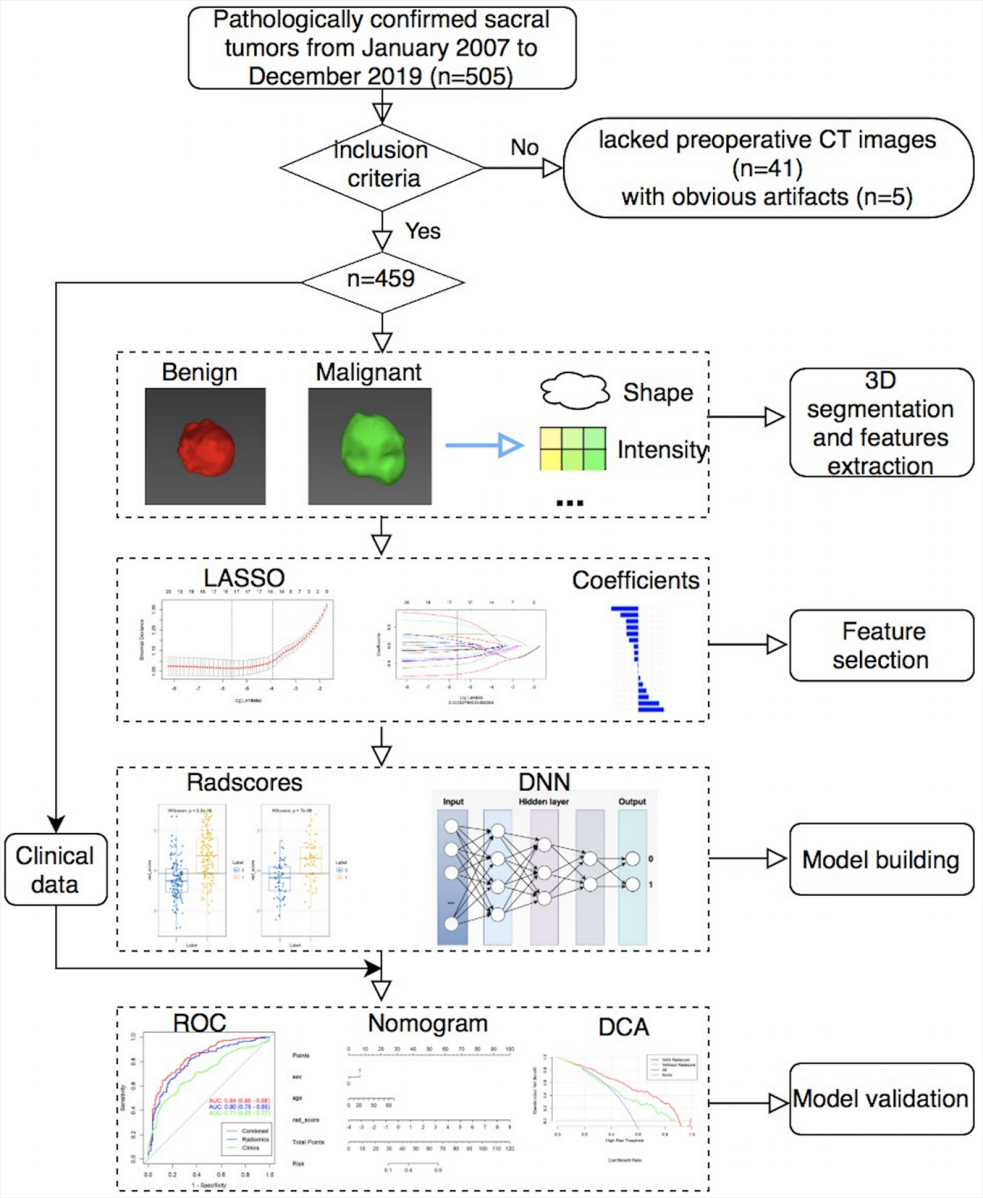
The workflow of this study.

The performance of different models was assessed using the area under the receiver operating characteristic curve (AUC). The accuracy (ACC), sensitivity, and specificity values were also reported for both the RMs and DNN model. Comparisons between AUCs were made by using DeLong test. The calibration curves and Hosmer–Lemeshow test were used to investigate the performance of the nomogram. The clinical usefulness of the nomogram was evaluated using decision curves analysis. **Figure 1** showed the workflow of this study.

### Statistical Analysis

Statistical analysis was performed on R software (R Core Team, Vienna, Austria) version 3.4.3. Mann-Whitney U test was performed to compare continuous variables, while chi-squared test was used for classify variables between groups. All statistical tests were two-sided, and a *P* value less than 0.05 was considered statistically significant.

## Results

### Patient Characteristics

A total of 459 patients (255 males, 204 females; mean age of 42.1 ± 17.8 years, range 4–82 years) were included in this study (**Table 1**). We found significant statistical differences in terms of sex, age and tumor size of patients with benign and malignant tumors (*P* < 0.01). There was a significant difference in the sex ratio between the two groups (*χ^2^* = 10.854, *P* = 0.001), in which the proportion of male patients with malignant tumors was significantly higher than that of female patients. The median age of benign tumor patients (38.0, in the range of 29.0–49.1) was significantly lower than that of the malignant tumor patients (53.0, 37.0–63.0) (*Z* = −6.616, *P* < 0.01). In addition, the size of the benign tumor was significantly larger than that of the malignant tumor (*Z* = 2.843, *P* < 0.01). Multivariable LR analyses showed that radscore, sex, and age (odds ratio [OR]1 = 2.492, OR2 = 2.236, OR3 = 1.037, *P* < 0.01) were important predictors of benign or malignant sacral tumors (**Table 2**).

**Table 1:** Clinical characteristic of patients.

Variable	Benign tumor	Malignant tumor	*χ^2^/Z* value	*P* value
Sex				
Female	109(52.91%)	95(37.55%)	10.854	0.001
Male	97(47.09%)	158(62.45%)		
Age (years)	38.00(29.00, 49.05)	53.00(37.00, 63.00)	−6.616	<0.001
Tumor size (cm)	8.60(6.70, 11.01)	7.90(5.90, 10.00)	2.843	0.004
Tumor type			–	–
Metastatic tumor	–	71(28.06%)		
Chordoma	–	84(33.20%)		
GCT	95(46.12%)	–		
Osteosarcoma	–	16(6.32%)		
Chondrosarcoma	–	20(7.91%)		
Schwannoma	47(22.82%)	–		
Neurofibroma	44(21.36%)	–		
Ewing’s sarcoma	–	28(11.07%)		
Multiple myeloma	–	15(5.93%)		
Other types	20(9.70%) ^a^	19(7.51%) ^b^		

GCT, giant cell tumor. a, the other types included 6 solitary fibromas, 3 ependymomas, 3 hemangiomas, 3 chondroblastomas, 3 aneurysmal bone cysts, 1 bone cyst, and 1 paraganglioma. b, the other types included 4 malignant teratomas, 5 lymphomas, 5 liposarcomas, 2 undifferentiated sarcomas, 1 synovial sarcoma, 1 epithelioid sarcoma, and 1 malignant granulosa cell tumor.

**Table 2:** Multivariable logistic regression analyses.

Intercept and variable	CT		
	Coefficient	OR (95% CI)	P
Intercept	−2.1372	–	0.0001
Radscore	0.9130	2.492 (1.937,3.206)	<0.0001
sex	0.8048	2.236 (1.3,3.848)	0.0036
age	0.0366	1.037 (1.02,1.054)	<0.0001
size	0.0122	1.012 (0.935,1.096)	0.7639

OR, odds ratio; CI, confidence interval.

No significant statistical difference was observed between the training and validation sets in terms of age, sex, and tumor location (*P* > 0.05). The 206 benign tumors were composed of 95 GCTs, 47 schwannomas, 44 neurofibromas, 6 solitary fibromas, 3 ependymomas, 3 hemangiomas, 3 chondroblastomas, 3 aneurysmal bone cysts, 1 bone cyst, and 1 paraganglioma. The 253 malignant tumors included 71 metastatic tumors, 84 chordomas, 16 osteosarcomas, 20 chondrosarcomas, 28 Ewing’s sarcomas, 15 multiple myelomas, 4 malignant teratomas, 5 lymphomas, 5 liposarcomas, 2 undifferentiated sarcomas, 1 synovial sarcoma, 1 epithelioid sarcoma, and 1 malignant granulosa cell tumor, respectively.

### Performance of Different Models

The reproducibility of radiomics features of different machines was satisfactory (ICC, ranged from 0.76 to 0.91).

**Figure 2:**
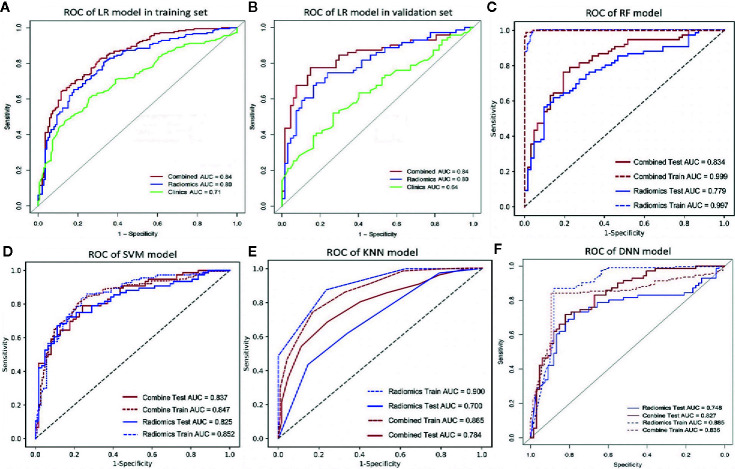
The ROC curve of different models. **(A, B)**, the ROC of LR-based clinical-RM in the training set **(A)** and validation set **(B)**. The blue line indicates radiomics model, the green line represents clinical model, and the red line is the LR-based clinical-RM; **(C–F)**, the ROC of RF-based clinical-RM **(C)**, SVM-based clinical-RM **(D)**, KNN-based clinical-RM **(E)**, and clinical DNN model **(F)**. The dotted blue line represents the RM **(C–E)** or DNN **(F)** model in the training set, and the solid blue line represents the RM **(C–E)** or DNN **(F)** model in the validation set. The dotted red line represents the clinical-RM **(C–E)** or clinical-DNN **(F)** model in the training set, and the solid blue line represents the clinical-RM **(C–E)** or clinical-DNN **(F)** model in the validation set.

Among the four RMs, RF had the best performance (AUC = 1, ACC = 0.98), followed by KNN (AUC = 0.90, ACC = 0.83), SVM (AUC = 0.85, ACC = 0.80) and LR (AUC = 0.80, ACC = 0.75) in the training set (**Figure 2**, **Table 3**). When combined with clinical features, a similar result was found; clinical-RF performed best, with an AUC value of 1 and an ACC value of 0.99.

**Table 3:** Performance of different models in training set and validation set.

	AUC	ACC	Sensitivity	Specificity	PPV	NPV
LR	0.80(0.80)	0.75(0.69)	0.81(0.76)	0.67(0.61)	0.76(0.68)	0.73(0.71)
RF	1(0.78)	0.98(0.72)	0.99(0.76)	0.95(0.66)	0.96(0.73)	0.99(0.70)
SVM	0.85(0.83)	0.80(0.75)	0.85(0.75)	0.74(0.76)	0.80(0.79)	0.80(0.71)
KNN	0.90(0.70)	0.83(0.64)	0.88(0.62)	0.76(0.66)	0.82(0.69)	0.83(0.59)
DNN	0.89(0.75)	0.88(0.72)	0.90(0.70)	0.84(0.74)	0.87(0.79)	0.88(0.64)
Clinics	0.71(0.64)	0.67(0.62)	0.76(0.66)	0.59(0.59)	0.61(0.54)	0.74 (0.70)
Clinical-LR	0.84(0.84)	0.75(0.81)	0.88(0.85)	0.65(0.78)	0.64(0.77)	0.88(0.85)
Clinical-RF	1(0.83)	0.99(0.77)	0.99(0.82)	0.99(0.71)	0.99(0.78)	0.99(0.76)
Clinical-SVM	0.85(0.84)	0.79(0.76)	0.83(0.76)	0.74(0.76)	0.80(0.80)	0.78(0.72)
Clinical-KNN	0.87(0.78)	0.78(0.72)	0.74(0.68)	0.83(0.76)	0.85(0.78)	0.72(0.66)
Clinical-DNN	0.84(0.83)	0.87(0.76)	0.91(0.80)	0.82(0.73)	0.85(0.72)	0.89(0.81)

AUC, area under curve; ACC, accuracy; PPV, positive predictive value; NPV, negative predictive value. Training set, in front of the brackets. Validation set, in brackets.

**Figure 3:**
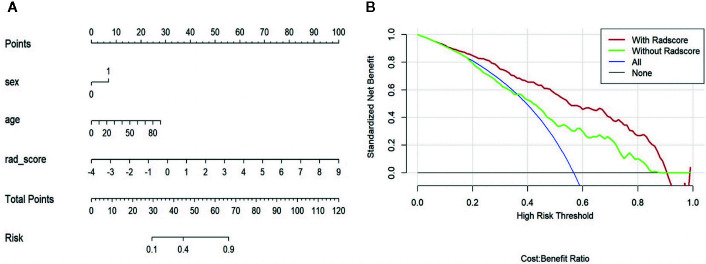
LR-based clinical-radiomics nomogram **(A)** and decision curves **(B)**. **(A)** The final total points were calculated by summing the score of each point represented for each feature. The nomogram showed that radscore was the most important factor. **(B)** The green line represents the clinical model. The red line represents the clinical-radiomics model. Decision curves showed that clinical-radiomics model achieved more clinical utility than clinical model.

In validating set, the performance of SVM (AUC = 0.83, ACC = 0.75) was the best among the four RMs, followed by LR (AUC = 0.80, ACC = 0.69), RF (AUC = 0.78, ACC = 0.72), and KNN (AUC = 0.70, ACC = 0.64). When combined with clinical features, however, clinical-LR had the best performance, with an AUC of 0.84 and an ACC of 0.81. Clinical-KNN performed the worst (AUC = 0.78, ACC = 0.72). Furthermore, clinical-RMs (AUC, ranged from 0.78 to 0.84; ACC, ranged from 0.72 to 0.81) performed better than individual RMs (AUC, ranged from 0.70 to 0.83; ACC, ranged from 0.64 to 0.75) and clinical model (AUC = 0.64, ACC = 0.62) in the validation set. **Figure 3** showed LR-based clinical-radiomics nomogram and decision curves.

The DNN model achieved an AUC of 0.75 and an ACC of 0.72 in the validation set. When combined with clinical data, the clinical-DNN model based on CT features exhibited an AUC of 0.84 and an ACC of 0.87 in the training set, and an AUC of 0.83 and an ACC of 0.76 in the validation set. In addition, no significant difference was found in terms of AUCs between the clinical-LR model and clinical-DNN model in the training (*P* = 0.889) and validation sets (*P* = 0.762).

## Discussion

In this study, we found that radscore, sex, and age were important indicators for differentiating benign and malignant sacral tumors. Among the four clinical-RMs, clinical-LR had the best performance in the validation set. The best-performing clinical-LR model exhibited an AUC of 0.84 and an ACC of 0.81 in the validation set. In addition, the clinical-DNN model also had a high performance in identifying benign and malignant sacral tumors. Our clinical-DNN and clinical-RMs would have a high impact on assisting radiologists in their clinical diagnosis of sacral tumors.

Patients with sacral tumor share many similar clinical symptoms and disease course, which increases the difficulty of preoperative diagnosis. In this study, we found that sex, age and tumor size were important indicators for differentiating benign and malignant sacral tumors. The size of the benign tumor was significantly larger than that of the malignant tumor. What’s more, the mean age of patients with sacral malignant tumors was higher than that of patients with benign tumors. The possible reason is that the largest proportion of patients with sacral malignant tumors are metastatic tumors and chordomas, which are most common in patients over 40 years old (2, 22). Furthermore, the proportion of males in patients with malignant tumors was higher than that in patients with benign tumors, with a significant statistical difference. The incidence of chordoma is higher in men than in women, which is consistent with previous study (10).

Previous studies have compared the performance of deep learning and radiomics in differentiating benign and malignant breast lesions (13, 15), predicting lymph node metastases of breast cancer (14), identifying of spinal metastases originated from the lung and other cancers (16), predicting of survival of patients with high-grade gliomas (17), and predicting the invasiveness risk of Stage-I lung adenocarcinomas (18). Dong et al. (23) recently compared the DNN model, LR and SVM to predict lymph node status in operable cervical cancer, and they also found that DNN performed best. Bibault et al. (24) found that their DNN model was 80% accurate in predicting complete response after neo-adjuvant chemoradiotherapy in locally advanced rectal cancer, which was better than LR and SVM models. Due to the rarity of primary sacral tumors, only a few previous studies have identified sacral tumor types using machine learning methods (1, 5, 10). In this study, we proposed a DNN model to identify benign and malignant sacral tumors. DNN has multiple hidden layers, which can extract features step by step, simplify problems and improve efficiency (12, 25). Song et al. (26) compared three types of DNN for classification of lung nodules on CT images. In this study, we trained four clinical-RMs and one clinical-DNN model based on a relatively large sample of data and found that clinical-LR performed best in the validation set. Similarly, Lang et al. (16) found that the accuracy of radiomics analysis and convolutional neural network (CNN) was similar in the identification of spinal metastases originated from the lung and other tumors. LR is one of the most commonly used algorithms in radiomics analysis and has been proved to be effective (27–30). Despite nomogram’s visualization, it has limited power for future big data era. On the contrary, deep learning is like a “black box”, its development trend is inevitable and more conducive to the analysis of big data (23). In this study, we found no significant difference in terms of AUCs between the clinical-LR and clinical-DNN models. Therefore, we still have no reason not to recommend the deep learning model. Our clinical-DNN model can also provide a convenient and accurate tool for radiologists to identify benign and malignant sacral tumors.

Our study has certain limitations. First, all images were collected from one center over the past decade or so. And we excluded some patients who did not have preoperative CT, which may lead to selection bias. A larger sample data from multicenter is needed in the further study to improve our models. Second, all images were obtained on the same type of plain CT scan. In the future, we will evaluate our models on more heterogeneous image data. Third, we only compared several common machine learning algorithms with DNN, and more algorithms (e.g., CNN) may be added in the future.

In conclusion, both the clinical-LR and clinical-DNN models could be used for assisting radiologists in their clinical diagnosis of sacral tumors.

## Data Availability Statement

All datasets presented in this study are included in the article/supplementary material.

## Ethics Statement

The studies involving human participants were reviewed and approved by: The local ethics committee of Peking University People’s Hospital. The requirement for written informed consent was waived due to the retrospective nature of this study.

## Author Contributions

1. Guarantor of integrity of the entire study: NH. 2. Study concepts: PY, NH. 3. Study design: PY, NH. 4. Definition of intellectual content: PY, HC. 5. Literature research: PY, HC. 6. Clinical studies: PY, NM. 7. Experimental studies: PY, NM, XL. 8. Data acquisition: PY, CS. 9. Data analysis: PY, SW. 10. Statistical analysis: SW, PY. 11. Manuscript preparation: PY, HC, NM. 12. Manuscript editing: PY. 13. Manuscript review: NH. PY, NM, and HC contributed equally to this study. All authors contributed to the article and approved the submitted version.

## Funding

This work was supported by National Key Research and Development Program of China (No. 2017YFC0109003).

## Conflict of Interest

Author SW was employed by the company GE Healthcare.

The remaining authors declare that the research was conducted in the absence of any commercial or financial relationships that could be construed as a potential conflict of interest.

## Abbreviations

GCT, Giant cell tumors; CT, Computed tomography; DNN, Deep neural network; LASSO, Least absolute shrinkage and selection operator; LR, Logistic regression; RF, Random forest; SVM, Support vector machine; KNN, k-nearest neighbor; ICC, intra- and interclass correlation coefficients; Radscore, Radiomics score; mRMR, minimum redundancy maximum relevance; AUC, Area under the receiver operating characteristic curve; ACC, Accuracy.
